# Neuropsychiatric involvement in juvenile-onset systemic lupus erythematosus (jSLE)

**DOI:** 10.1186/s40348-023-00161-7

**Published:** 2023-08-09

**Authors:** Valentina Natoli, Amandine Charras, Gabriele Hahn, Christian M. Hedrich

**Affiliations:** 1 Department of Women’s and Children’s Health, Institute of Life Course and Medical Sciences, University of Liverpool, Liverpool, UK; 2 Department of Rheumatology, Institute in the Park, Alder Hey Children’s NHS Foundation Trust, Liverpool, UK; 3 Dipartimento di Neuroscienze, Riabilitazione, Oftalmologia, Genetica e Scienze Materno-Infantili, Università degli Studi di Genova, Genoa, Italy; 4 Department of Radiology, Universitätsklinikum Carl Gustav Carus, Dresden University of Technology, Dresden, Germany

**Keywords:** SLE, jSLE, NP-jSLE, Juvenile, Lupus, Neuropsychiatric, Neurologic, Treatment, Inflammation, Interferon, CNS

## Abstract

Systemic lupus erythematosus (SLE) is a rare autoimmune/inflammatory disease with significant morbidity and mortality. Approximately 15–20% of SLE patients develop the disease during childhood or adolescence (juvenile-onset SLE/jSLE). Patients with jSLE exhibit more variable and severe disease when compared to patients with disease-onset during adulthood. Neuropsychiatric (NP) involvement is a clinically heterogenous and potentially severe complication. Published reports on the incidence and prevalence of NP-jSLE are scarce, and the exact pathophysiology is poorly understood.

This manuscript provides a review of the existing literature, suggesting NP involvement in 13.5–51% of jSLE patients. Among patients with NP-jSLE affecting the CNS, we propose two main subgroups: (i) a chronic progressive, predominantly type 1 interferon-driven form that poorly responds to currently used treatments, and (ii) an acutely aggressive form that usually presents early during the disease that may be primarily mediated by auto-reactive effector lymphocytes. While this hypothesis requires to be tested in large collaborative international cohort studies, it may offer future patient stratification and individualised care.

## Introduction

Systemic lupus erythematosus (SLE) is a severe and potentially life-threatening chronic autoimmune/inflammatory disease [1–3]. It can affect any organ system of the human body and cause significant damage resulting in organ failure. Despite recent progress, the complex molecular pathophysiology of SLE remains incompletely understood. Genetic factors play a pronounced role; however, only relatively few SLE patients experience “purely” genetic disease caused by mutations in single genes. The majority of SLE patients exhibit a genetic predisposition that requires the accumulation of additional, environmentally mediated factors to develop the disease [3–9].

Genetic factors are associated with variable incidences and disease severity between ethnicities and age groups. The highest incidence and prevalence of SLE worldwide is seen in North America [23.2/100,000 person-years (95% CI 23.4–24.0) and 241/100,000 people (95% CI 130–352)], respectively, while the lowest incidences are observed in Africa and Ukraine (0.3/100,000 person-years). Access to health care and missed diagnoses may play a role in some regions (e.g., in rural Africa). Indeed, in Western countries, SLE cohorts are characterised by a significant over-representation of Black African/Caribbean ethnicities and East Asians. In the UK, the incidence is reported to range around 5 cases/100,000 person-years [3, 10, 11].

Approximately 15–20% of patients develop SLE before their 16th birthday and are therefore diagnosed with juvenile-onset systemic lupus erythematosus (jSLE) [12, 13]. Notably, when compared to disease-onset in adulthood, jSLE is associated with higher and more wide-spread organ involvement at diagnosis, increased organ damage, and increased need for anti-inflammatory/immune-modulating treatments including corticosteroids [1, 2, 4, 8, 14–16]. Among children (and adults), minority ethnicities are most frequently and severely affected, and preliminary studies linked a high number of risk alleles in an individual with early disease-onset, increased clinical disease activity and severity, as well as Black African ethnicity [5, 9, 17].

Neuropsychiatric SLE (NP-SLE) is a potentially severe and sometimes life-threatening complication of SLE [18, 19]. It can significantly impact on patients’ quality of life, disease outcomes and prognosis. Notably, NP involvement associates with an estimated three-fold increase in mortality when compared to SLE patients without NP disease [20].

Neuropsychiatric features in jSLE are extremely heterogeneous, which is reflected by 19 items suggested by the American College of Rheumatology (ACR), including such related to both the central (CNS) and/or the peripheral (PNS) nervous system (Table 1) [21]. Therefore, diagnosis and treatment of NP-jSLE should be guided by experienced multidisciplinary teams. Notably, even with such a broad range of manifestations included, the ACR classification may not cover all presentations. Notably, neuromyelitis optica spectrum disorders, posterior reversible encephalopathy syndrome, leukoencephalopathy, and chronic inflammatory demyelinating polyneuropathy are not included specifically in this classification [22–25]. Lastly, fatigue is a common concern among patients with SLE across age groups that significantly affects their quality of life and wellbeing [13]. However, it can be difficult (or even impossible) to answer the question of whether fatigue is caused by NP involvement or a not specific “constitutional” symptom of inflammation or associated with "low mood” [26, 27].

**Table 1:** American College of Rheumatology case definitions for neuropsychiatric SLE (1999) [21]

**Central nervous system (CNS)**	
Diffuse	Acute confusional state
	Anxiety disorder
	Aseptic meningitis
	Cognitive dysfunction
	Demyelinating syndrome (Fig. 1A)
	Headache
	Mood disorder
	Psychosis
Focal	Cerebrovascular disease (Fig. 2)
	Movement disorder
	Myelopathy (Fig. 1B)
	Seizures
**Peripheral nervous system (PNS)**	
Acute inflammatory demyelinating polyradiculoneuropathy (Guillain-Barré syndrome) (Fig. 1C)	
Autonomic disorder	
Cranial neuropathy	
Mononeuropathy	
Myasthenia gravis	
Plexopathy	
Polyneuropathy	

Identification and diagnosis of NP features in jSLE patients can be challenging, not only because of the range of clinical presentations, but also secondary to the relatively high prevalence of symptoms not specific to jSLE, such as headaches, mood and anxiety disorders, and catatonia [19]. In fact, the attribution of NP manifestations to SLE-related causes is as complex as critical. It is currently guided by experience-based criteria, such as the interval between the diagnosis of jSLE and the NP event, the prevalence of NP events in non-SLE individuals and the presence of other risk factors for NP involvement not related to jSLE (e.g., ongoing infections, metabolic anomalies) [28].

To date, relatively few studies with limited patient numbers focused on NP-jSLE [19, 29–34]. Based on these and following the general trend, NP-SLE may be more common and aggressive in jSLE (15–95%) when compared to adult-onset SLE (14–80%) [19, 30–35]. Considering psychosocial aspects, morbidity and mortality associated with NP disease, timely diagnosis, outcome prediction and informed individualised treatment of NP-SLE are critical [36].

### Presentation and diagnosis

Neuropsychiatric involvement in jSLE varies in its presentation and severity, ranging from mild symptoms such as mild headaches or anxiety disorders, to life-threatening manifestations [14, 16, 18, 19, 30–34, 37, 38]. While some studies suggested that most patients exhibit NP at diagnosis [39], several studies reported that 30–70% of jSLE patients develop NP involvement more than 6 months after diagnosis [19, 31, 32].

Across studies in jSLE, most NP manifestations reported relate to the CNS (Table 2). Differences between cohorts may be associated with the inclusion of patients with headaches as a singular symptom, which were excluded in recent studies accessing the UK jSLE Cohort and Turkish patients. Across studies, headaches were the most frequently observed symptom of NP-jSLE (36–78.5%) patients. Notably, when considering data from the UK, among jSLE patients with no NP involvement, headaches were also common (177/321, 55.1%) [19, 30–34]. Migraines represent the most common type of headache associated with NP-jSLE. Notably, lupus headaches, which have been defined as severe and persistent headaches not responsive to narcotics and included in the SLEDAI scoring system as a marker of disease activity, are extremely rare [29].

**Table 2:** Juvenile-onset SLE with neuropsychiatric involvement

	Giani et al. [19]	YU et al. [32]	Kısaarslan et al. [31]	Khajezadeh et al. [30]	Zambrano et al. [33]	Singh et al. [34]
**Country of study**	UK	China	Turkey	Iran	Colombia	India
**Cohort size**						
**• Total jSLE patients, *N***	428	185	1107	146	90	53
**• NP-jSLE patients, *N* (%)**	107 (25)	64 (34.6)	149 (13.5)	41 (28)	30 (33.3)	27 (50.9)
**Female to male ratio**	5.4:1	5.9:1	7.7:1	3:1	5.4:1	2.8:1
**Mean age (SD)**	12.2 (± 3.1)	13.2 (± 3)	12.8	10.2 (± 3)	12.2	9.9 (± 3.2)
**Distribution of NP features (%) among NP-jSLE patients**						
**Central NS**	93	97	94.6	99.3	87	94.4
**Peripheral NS**	7	3	5.4	0.7	13	5.6
**Aseptic meningitis**	1.8	0	0.7	0.7	N/A	3.7
**Acute confusional state**	6.5	12.5	33.6	N/A	N/A	7.5
**Acute inflammatory demyelinating polyradiculoneuropathy (GBS)**	1	0	2	N/A	6	N/A
**Autonomic disorders**	2.8	1.6	2	N/A	N/A	N/A
**Anxiety disorders**	23.3	6.3	32.9	N/A	N/A	1.8
**Cerebrovascular disease**	14.9	39	14.8	5.3	26	11.3
**Cognitive dysfunction**	42	0	22.8	11.6	N/A	17
**Cranial neuropathy**	5.6	1.6	3.4	0.7	N/A	1
**Demyelinating syndrome**	1.8	3.1	2	N/A	N/A	1.8
**Headache**	78.5	10.9	50	13	36	39.6
**Movement disorders**	17.7	0	16.8	3.4	16	5.6
**Mood disorders**	48.6	12.5	29.5	5.4	N/A	9.4
**Myelopathy**	0.9	4.7	2.7	N/A	N/A	N/A
**Psychosis**	9.3	21.9	17.4	2.1	13	9.4
**Seizures**	19.6	84.4	38.3	9.5	50	35.8
**Mononeuropathy**	8.4	3.1	2	N/A	N/A	N/A
**Myasthenia gravis**	0	0	0	N/A	N/A	N/A
**Plexopathy**	0.9	0	0	N/A	N/A	N/A
**Polyneuropathy**	1.8	4.7	6	N/A	N/A	N/A

Only studies with sufficient clinical and demographic data have been included

*jSLE* juvenile systemic lupus erythematosus, *NP* neuropsychiatric, *UK* United Kingdom, *SD* standard deviation, *NS* nervous system, *GBS* Guillain-Barré syndrome, *N/A* not applicable

Common neuropsychiatric features of jSLE furthermore include seizures (9.5–84.4%) that are frequently associated with the presence of antiphospholipid antibodies (aPL), antiphospholipid syndrome, arterial hypertension, and other NP disorders, such as cerebrovascular disease. JSLE patients who experience seizures also have an increased risk of long-term neurological damage, including neurocognitive dysfunction and epilepsy [40]. Cognitive dysfunction (11.6–42%) and mood disorders (5.4–48.6%) are also highly prevalent. Psychotic episodes have been reported across jSLE cohorts (9–22% of NP-jSLE patients) and can result in missed diagnosis and/or admission to psychiatric units [19, 30–34].

New onset of any of these symptoms requires careful consideration of infectious and/or cerebrovascular causes. Cerebrovascular disease in jSLE (5.3–39%) associates with various cerebral blood vessel abnormalities that range from vasculitis affecting small, medium or large arteries to cerebral vein thrombosis. Notably, cerebral venous thrombosis in almost all cases is associated with the presence of aPL antibodies, especially lupus anticoagulant [41]. Cerebrovascular disease represents an acute medical emergency that requires immediate attention and aggressive anti-inflammatory treatment to prevent vessel occlusion and/or haemorrhage [19, 30–34]. Because of their rarity and likely underdiagnosis, data on peripheral nervous system (PNS) involvement are even more limited. An estimated 5–15% of jSLE patients and develop either mono- or polyneuropathy that can affect both sensory and motor neurons. Cranial neuropathy (2.7%), myasthenia gravis (1.3%), acute inflammatory demyelinating polyradiculoneuropathy (Guillain-Barré syndrome [GBS]) (2.5%) and autonomic neuropathy are extremely rare (1.9%) [38]. The onset of neuropathy usually correlates with high disease activity [42].

As mentioned above, in some studies, > 50% of patients developed NP involvement over time, and > 6 months after a diagnosis of SLE was established [19, 31, 32]. Notably, in the UK jSLE cohort, when compared to patients with “early” NP involvement, “late” development of NP features was associated with higher proportions of patients with persisting “severe” or “mild” disease activity (British Isles Lupus Assessment Group (BILAG) NP domain) and a reduced proportion of patients with inactive neurologic disease at last visit [19]. This is in line with reports on a sub-cohort of SLE patients from the UK jSLE Cohort Study (*N* = 348 total) with “genetic forms” of jSLE (*N* = 12), who exhibited lower disease activity at diagnosis but increased activity at their last visit when compared to the remaining jSLE cohort (*N* = 336) [5]. Increased disease activity scores were caused by the development of neuropsychiatric features over time, usually in the absence of haematologic or renal involvement, two manifestations that associate with classical SLE phenotypes and the presence of high-titre autoantibodies. This suggests that patients with genetic forms of SLE may not only exhibit distinct pathophysiological features but also differential disease outcomes that may likely be caused by the pathological activation of innate antiviral responses (type 1 interferon-associated gene expression) that may be addressed by patient stratification towards individualised care (see below).

Diagnosing NP-jSLE can be challenging because of the lack of sensitive and disease-specific tests [43]. Indeed, there are no specific guidelines for the diagnostic workup of patients with NP-jSLE. The European Single Hub and Access Point for Paediatric Rheumatology in Europe (SHARE) recommendations [44] remained cautious and vague in relation to NP-jSLE diagnosis and treatment and suggested the exclusion of alternative diagnoses and/or jSLE-associated organ complications [37]. Initial assessment in patients with NP symptoms includes evaluation of systemic disease activity, exclusion of infections (blood and cerebrospinal fluid (CSF) cultures), metabolic alterations, arterial hypertension and adverse drug reactions as underlying causes of symptoms.

**Fig. 1:**
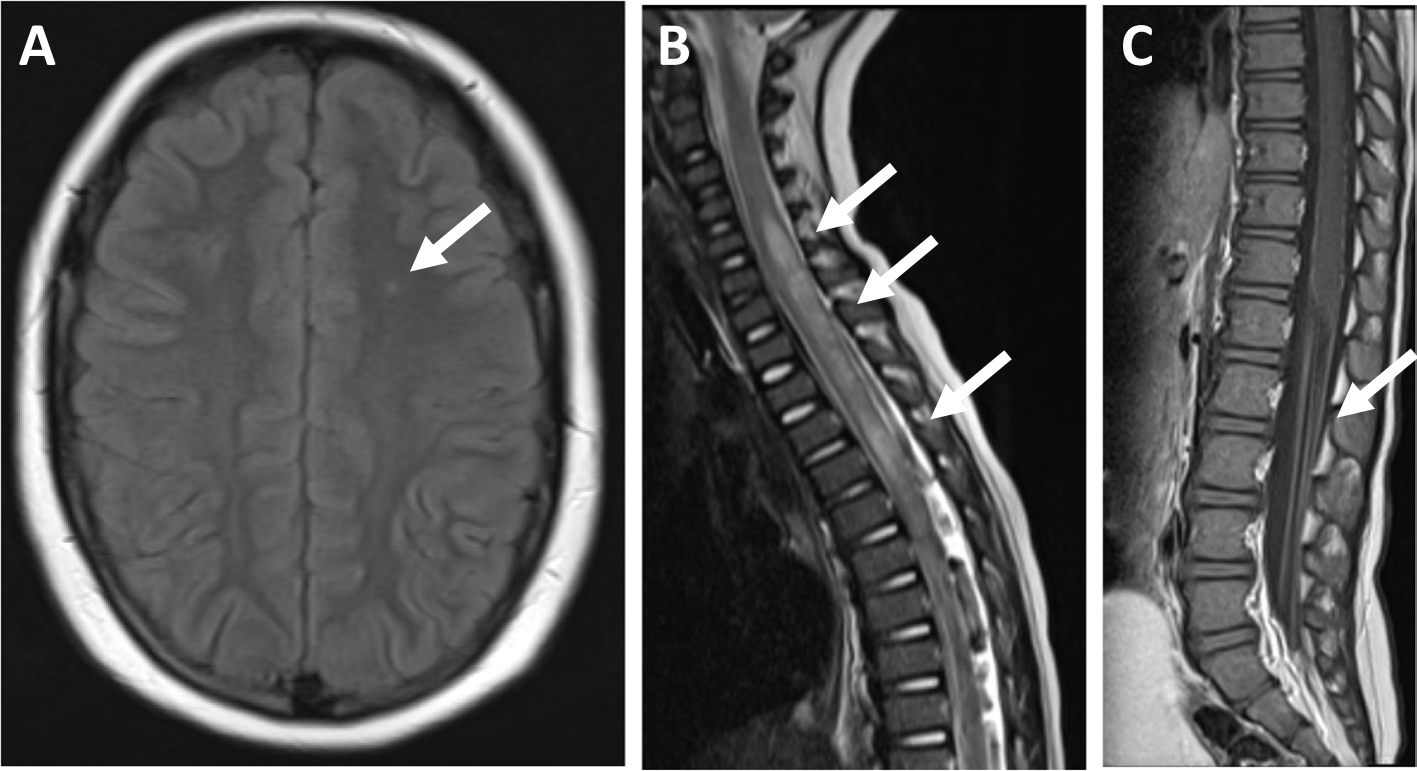
Immune-mediated neurological disorders in jSLE. **A** Demyelinating syndrome (DS) in a 15-year-old patient. Transversal MRI images showing increased spotty signals in frontal white matter in Fluid attenuated inversion recovery (FLAIR) sequences. **B** Myelopathy in a 10-year-old patient. Sagittal MRI with high-signal of the myelin in T2-weighted images. **C** Guillain-Barré syndrome in a 12-year-old patient. Contrast enhancement of the cauda equina; sagittal T1-weighed images

**Fig. 2:**
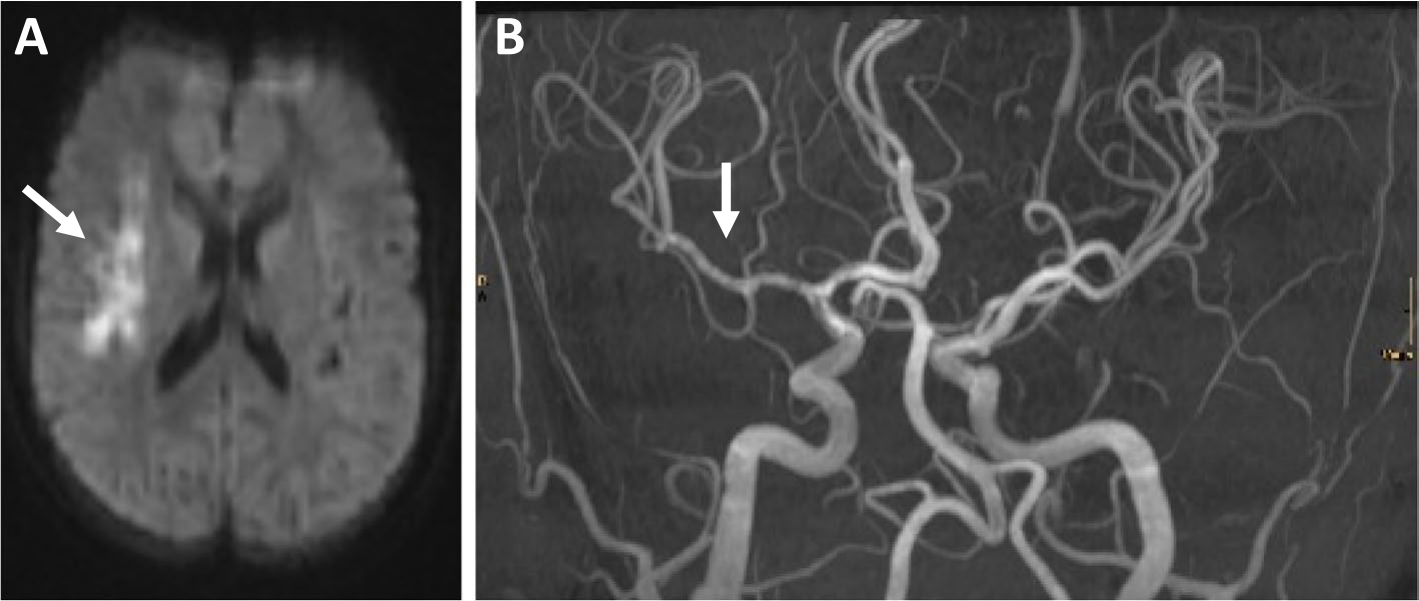
Cerebrovascular involvement in jSLE. **A** Infarction in a 15-year-old patient. Transversal MRI with diffusion disorder in right temporal lobe; DWI sequences. **B** Vasculitis of the *arteria cerebri media* with vascular irregularities in MR angiography (MRA)

Magnetic resonance imaging (MRI) is currently the most widely used imaging modality for investigating NP-jSLE (Figs. 1 and 2). While MRI is fundamental to rule out alternative diagnoses, there are no specific neuro-radiological findings that are definitive for NP-jSLE, and imaging can be normal, even in cases with small vessel CNS vasculitis. A single-centre study investigating 27 NP-jSLE patients (22 girls; median age 11 years (4–15) reported normal MRI in 59%, followed by focal white matter anomalies (in T2-weighted sequences) (33%), brain atrophy (18.5%), basilar artery infarction (3%) and cortical grey matter lesions (3%). Notably, the presence of anxiety is associated with abnormal MRI findings (*p* = 0.008) [45]. Evaluation of CSF for inflammatory markers, including white blood cell count, protein, oligoclonal bands and the Immunoglobulin G (IgG)/albumin CSF index, is commonly performed. Nevertheless, there is little evidence that these markers are significantly sensitive or specific for NP-jSLE. Currently available blood or CSF biomarkers include aPL antibodies, serum anti-ribosomal P protein antibodies and anti-N-methyl-d-aspartate receptor subunit 2 (NR2) antibodies [46, 47]. A recent study suggested that interferon (IFN)-α and neopterin levels in the CSF may represent useful diagnostic and disease activity markers in NP-jSLE with CNS inflammation [48].

### Treatment options and pathomechanisms

The exact molecular pathophysiology of NP-SLE is unknown [49]. Several features, including headaches, mood disorder and psychosis may be triggered by systemic inflammation and autoantibodies penetrating the CNS [49, 50]. Cerebrovascular disease may be promoted by thrombocyte pathology and/or effector T cells [49, 51–53].

As a result of the limited understanding of cellular and molecular pathomechanisms, the treatment of NP-jSLE is empiric and not guided by controlled trials in either children or adults. This makes the management of NP-jSLE challenging for all, patients, families and care providers. The lack of evidence from prospective trials and limited reports are reflected by the cautious wording of the European evidence and consensus-driven SHARE initiative that suggested the use of corticosteroids and immunosuppressive drugs as indicated [44]. Thus, in clinical practice, treatment decisions are frequently guided by evidence from other diseases with similar features, expert opinion and experience within the centre.

**Fig. 3:**
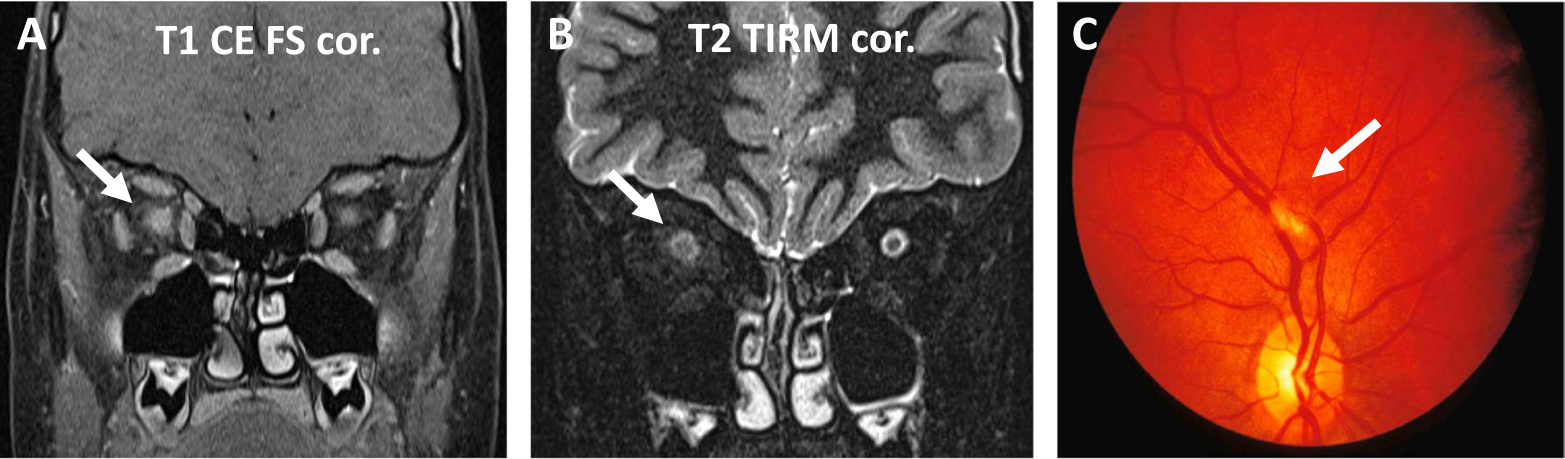
Neuromyelitis optica and retinitis in an 8-year-old jSLE patient. **A** Coronal MRI images showing perineural contrast enhancement of the right optic nerve in T1-weighted sequences (arrow). **B** Increased T2-weighted signal of the *Nervus opticus* and reduced signal of the nerve sheath (arrow) representing focal oedema of nerve and optic perineuritis. **C** Cotton-wool infiltrates resembling retinitis of the right eye in the same patient. The patient failed to respond to treatment with CPM and high-dose corticosteroids and was therefore treated with B cell depletion (rituximab), followed by repeated/cyclic immune-apheresis

If not caused by other complications of SLE (arterial hypertension, thromboembolism, infection, etc.), headaches frequently respond to analgesic treatments usually effective for other (severe) forms of chronic headaches [54, 55]. While data are limited, for the treatment of acute psychosis, antipsychotic medications should be considered and are frequently combined with cyclophosphamide (CPM). Indeed, patients enrolled in the UK jSLE cohort study had a >3-fold increased risk of receiving CPM when they had NP features (“only” 1.6-fold with renal disease) [56]. However, whether CPM is necessary or not remains unknown due to the lack of rigorous randomised clinical trials. Furthermore, the use of B cell depletion with rituximab (targeting CD20^+^ B cells, including late pre-B lymphocytes, B cells but not terminally differentiated plasmablasts and plasma cells) or belimumab (anti-B-cell activating factor (BAFF)/B-lymphocyte stimulator (BLyS)) alone or in combination with CPM has been discussed to treat NP-SLE. However, data are preliminary and limited to individual case reports or (small) case series in the adult-onset SLE population [57–60]. Recently, one study explored the depletion of long-lived plasma cells with the proteasome inhibitor bortezomib in a small otherwise treatment-refractory NP-jSLE cohort (5 patients) [61]. Notably, depletion of CD38^+^ plasma cells with the monoclonal antibody daratumumab (https://clinicaltrials.gov/ct2/show/NCT04810754) [62] or CD20^+^ B lymphocytes (spanning from pro-B cells to memory B cells) with the humanised antibody obinutuzumab [63, 64] are currently explored in adults with otherwise treatment-refractory SLE and may be future options also in patients with NP-jSLE. While not reaching statistical significance, in the UK jSLE cohort study, B cell depletion with rituximab was the second most frequently used immunomodulating treatment for NP-jSLE after CPM (OR 1.8, *p* = 0.3) [56]. Maintenance treatment usually includes azathioprine (AZA) or mycophenolate mofetil (MMF) [65]. In the absence of evidence from controlled trials, acute demyelination, transverse myelitis and/or neuromyelitis may be treated with high-dose corticosteroids, CPM, immune apheresis, and/or B cell depletion followed by maintenance immunomodulation (e.g., MMF) (Fig. 3) [61, 66, 67].

**Fig. 4:**
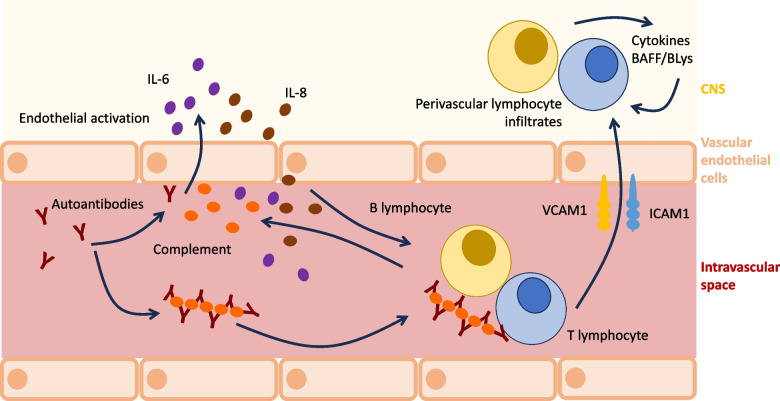
Proposed pathomechanisms of CNS vasculitis in NP-jSLE. Several, likely connected pathways may be involved in the pathogenesis of vasculitis in NP-jSLE. Circulating autoantibodies can recruit to vascular epithelia and/or form immune complexes that contribute to complement activation. Both, antibody binding and the presence of activated complement contribute to endothelial activation that, in turn, mediates the upregulation of adhesion molecules (including intercellular adhesion molecule 1/ICAM1 and vascular cell adhesion molecule 1/VCAM1) and the expression of pro-inflammatory cytokines, including IL-6 and IL-8. As a result, B and T lymphocytes are attracted, adhere to ICAM1/VCAM1 and infiltrate the perivascular region where they produce inflammatory cytokines and B cell survival factors (BAFF/BLys) [49]

As mentioned above, cerebrovascular disease as a manifestation of NP-jSLE usually includes CNS vasculitis (Fig. 2), thromboembolic events (e.g., in the context of antiphospholipid syndrome or as a result of vessel stenosis) and/or cerebral haemorrhage (e.g., in the context of CNS vasculitis; differential diagnosis: hypertensive crisis) [19, 29–34, 39, 68]. The molecular and cellular pathophysiology of NP-jSLE-associated CNS vasculitis is poorly understood but probably involves effector lymphocytes and autoantibodies (Fig. 4) [49]. As is the case for primary CNS vasculitis, also in NP-jSLE small vessel disease can be differentiated from vasculitis affecting medium to large vessels [53, 69, 70]. Except for some forms of primary medium/large vessel vasculitis (namely, transient angiography positive primary angiitis of the CNS/pPACNS), it appears unlikely that jSLE-associated vasculitis is self-limited and only requires short-term immunomodulating treatment with corticosteroids without the introduction of maintenance therapy. Thus, treatment of CNS vasculitis in NP-jSLE will, in most cases, includes high-dose corticosteroids (intravenous methylprednisolone/IVMP) and CMP, followed by a corticosteroid tapering regimen over 6–12 months and maintenance immunomodulation with MMF or AZA [53, 69, 70]. Thrombocyte aggregation inhibitors, e.g., acetylsalicylic acid, clopidogrel or their combination, should be discussed and maintained for a minimum of 12 months, guided by clinical symptoms, vessel morphology and perfusion (e.g., angio-MRI and diffusion-weighted imaging (DWI) sequences) [53, 69, 70].

Notably, although data are not available for all studies, severe NP features were frequently already present at diagnosis, including psychiatric symptoms, seizures and cerebrovascular disease [19, 31, 32]. This, together with associations with elevated C-reactive protein (CRP) and thrombocytopenia [19], suggests that uncontrolled inflammation, high autoantibodies titres and effector T lymphocyte populations may play a pronounced role [71–75]. Response to aggressive anti-inflammatory treatment with IVMP and CPM supports this hypothesis [19, 29, 56, 68]. This may be reflected by elevated levels of biomarker candidates of CNS inflammation, including IFN-α and neopterin in the CSF, which decrease after the induction of remission [48].

As mentioned above, not all patients exhibit NP symptoms within 6 months of diagnosis [19, 31, 32]. In the UK jSLE cohort study, patients with “genetic forms” of jSLE that can be explained by single gene mutations did not experience neuropsychiatric symptoms at diagnosis or early in disease, but developed neuropsychiatric involvement over time, resulting in higher disease activity in this sub-cohort of patients at their last visit, usually at transition to adult services [5]. The majority of “monogenetic” disease (75%) was caused by variants affecting type 1 IFN expression directly or indirectly (Table 3). This suggests that (i) NP disease (especially affecting the CNS) developed despite ongoing “standard” immunomodulating treatment, but (ii) may also be prevented using alternative approaches, e.g., targeting type 1 IFN signalling cascades. Notably, the aforementioned study reporting elevated IFN-α in the CSF of NP-jSLE patients (that, across a NP-jSLE patient cohort reduced after the induction of remission) did not discriminate between “genetic” versus “classic” forms of jSLE [48]. It appears intriguing to speculate that in “genetic” jSLE characterised by defects in type 1 interferon and complement pathways, CSF IFN-α levels may remain elevated when compared to healthy individuals, even after clinical improvement in response to treatment and achievement of clinical remission.

**Table 3:** Presumably damaging variants identified in the UK jSLE cohort [5]

**Gene variants**	**Predicted effect (snpEFF, ClinVar, snpNexus and PubMed)**	**Zygosity**	**Predicted impact**	**Reported inheritance patterns**	**Reference (first reported)**
*C1S* (rs117907409)	Deleterious	Heterozygous	Disease-causing	AD	[76]
*SAMHD1* (c.811C>A)	Impacts catalytic activity	Heterozygous	Disease-causing or -modifier	AD/AR	[77]
*C3* (rs117793540)	Gain-of-function	Heterozygous	Disease-causing	AD/AR	[78, 79]
*TREX1* (rs1331920811)	Loss-of-function	Heterozygous	Disease-causing or -modifier	AD/AR	[80]
*SAMHD1* (c.811C>A)	Impacts catalytic activity	Heterozygous	Disease-causing or -modifier	AD	[77]
*IRF7* (c.-283-1G>T)	Loss-of-function	Heterozygous	Disease-modifier	AR	—
*C3* (c.4341C>A)	Loss-of-function	Heterozygous	Disease-causing or -modifier	AD/AR	—
*RNASEH2B* (c.184G>T)	Deleterious	Heterozygous	Disease-modifier	AR	—
*BANK1* (rs928173624)	Loss-of-function	Heterozygous	Disease-causing or -modifier	N/A	[81, 82]
*PTPN22* (c.369+1G>T)	In silico predicted ‘high impact’	Heterozygous	Disease-causing	AD	—
*TNFAIP3* (rs776714084)	Loss-of-function	Heterozygous	Disease-causing	AD	—
*RNASEH2A* (rs549586181)	Loss-of-function	Heterozygous	Disease-modifier	AR	[80]
*C3* (c.1303G>T)	Loss-of-function	Heterozygous	Disease-modifier	AD/AR	—
*TNFSF4* (c.368_369delAG)	Loss-of-function	Heterozygous	Disease-modifier	N/A	—
*RNASEH2C* (rs759118175)	Loss-of-function	Heterozygous	Disease-modifier	AR	—
*TREX1* (rs749323787)	Impacts catalytic activity	Heterozygous	Disease-causing	AD/AR	[82]
*SAMHD1* (rs1400380009)	Loss-of-function	Heterozygous	Disease-causing	AD/AR	—
*PEPD* (rs529315200)	Loss-of-function	Heterozygous	Disease-modifier	AR	[83]
*DNASE1* (rs201571412)	Loss-of-function	Heterozygous	Disease causing	AD	[84]

*AD* autosomal dominant, *AR* autosomal recessive, *BANK1* B cell scaffold protein with ankyrin repeats 1, *C1S* complement component C1S, *C3* complement C3, *DNASE1* deoxyribonuclease 1, *IRF7* interferon regulatory factor 7, *N/A* not available, *PEPD* peptidase D, *PTPN22* protein tyrosine phosphatase non-receptor type 22, *RNASEH2A* ribonuclease H2 subunit A, *RNASEH2B* ribonuclease H2 subunit B, *RNASEH2C* ribonuclease H2 subunit C, *SAMHD1* SAM and HD domain containing deoxynucleoside triphosphate triphosphohydrolase 1, *TNFAIP3* TNF-α-induced protein 3, *TNFSF4* TNF superfamily member 4, *TREX1* three prime repair exonuclease 1

**Fig. 5:**
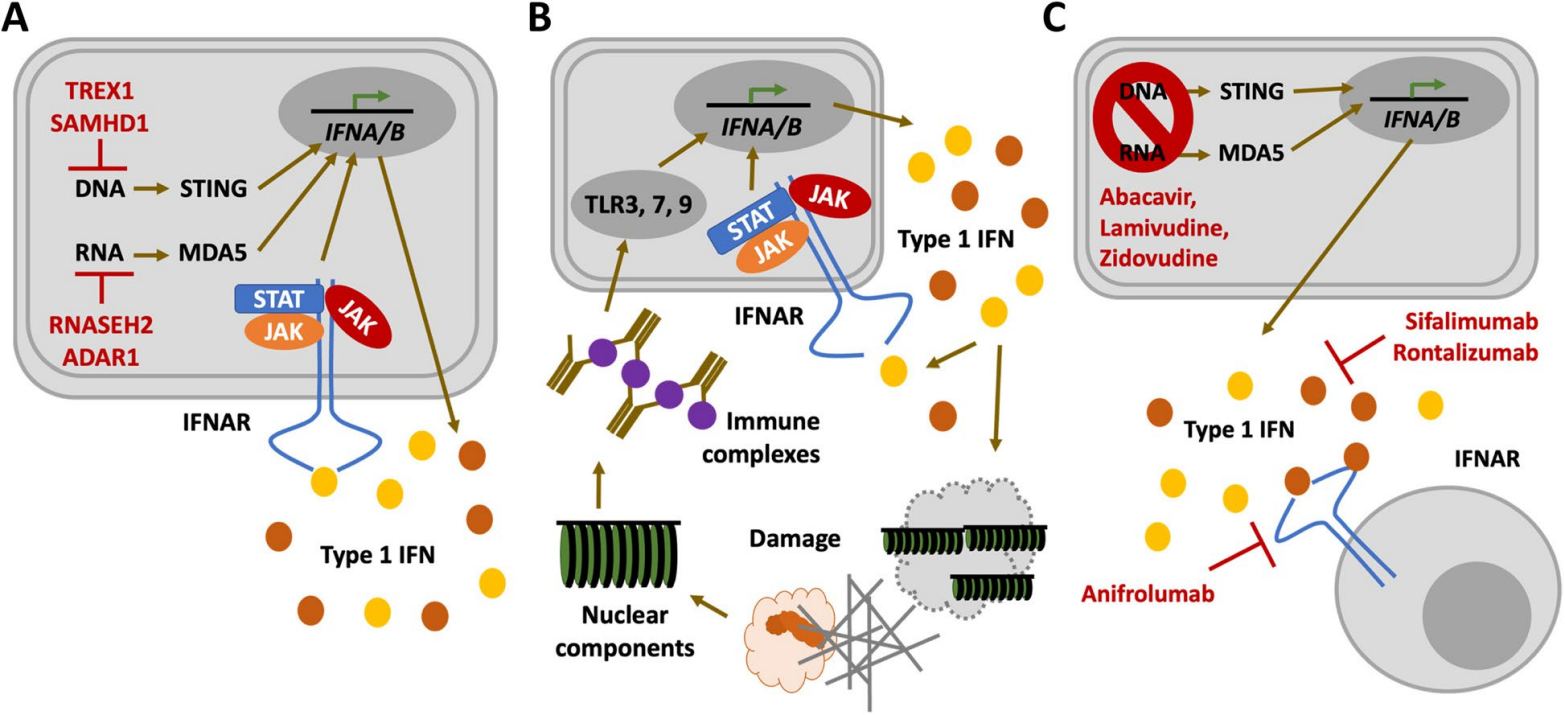
Molecular mechanisms of type 1 interferon production in jSLE. **A** In primary-type 1 interferonopathies, reduced metabolization/removal of cytoplasmic nucleic acid (e.g., through TREX1, SAMHD1, RNASEH2, ADAR1) or increased/spontaneous activation of cytoplasmic nucleic acid sensors (e.g., STING, MDA5) result in increased type 1 IFN expression (*IFNA/B*) and release. Release of type 1 interferons results in binding to and activation of type 1 IFN receptors (IFNAR), a mechanism that amplifies IFN expression through the Janus Kinase (JAK) signal transducer and activator of transcription (STAT) pathway. **B** In secondary type 1 interferonopathies but also “classic” SLE, deposition of immune complexes results in their detection by Toll-like receptors (namely TLR3, 7, 9), which triggers type 1 IFN expression and release. Interferons enhance their own expression through the JAK/STAT pathway and cause tissue damage through immune activation and the propagation of inflammation. This results in the release of cellular components and the generation of autoantibodies, which amplifies immune complex formation and deposition. **C** Type 1 IFN signalling can be inhibited using antiretroviral drugs (e.g., abacavir, lamivudine, zidovudine), IFN-I antibodies (sifalimumab, rontalizumab), or inactivating type 1 IFN receptor antibodies (anifrolumab)

Most patients with “genetic forms” of jSLE exhibit mutations in the complement cascade or genes involved in the detection and removal of nucleic acids from the cytoplasm of the extracellular compartment (Fig. 5A, B) [4, 5, 82]. These two pathways are closely connected and, together, contribute to pathological auto-amplification of inflammation [85]. Complement defects affecting the classical activation cascade (namely C1, C2, C3 and C4) are characterised by increased deposition of immune complexes and, as a result, type 1 IFN expression following Toll-like receptor (TLR) activation. Furthermore, a recent study on stress-induced neuroinflammation identified C3 as a key regulator of linking type 1 IFN expression in the CNS of humans and mice [86]. Complement defects may therefore be classified as “secondary type 1 interferonopathies” [4, 8, 87, 88]. “Primary type 1 interferonopathies”, on the other hand, are characterised by spontaneous and uncontrolled expression of interferon-stimulated genes (ISGs) [89]. Interferon production promotes inflammation and associated tissue damage, thereby contributing to the release of nuclear antigens and the generation of autoantibodies and immune complexes [87]. Therefore, inhibition of type 1 IFN signalling, e.g., with Janus kinase (JAK) inhibitors, has been considered [90–92], and recent clinical trials in adult SLE patients show promising results (e.g., applying tofacitinib and baricitinib), but the information in jSLE remains limited [93].

Some jSLE-associated mutations have previously been reported in a neuroinflammatory disease that shares systemic features with SLE and other autoimmune/inflammatory diseases, i.e., Aicardi-Goutières syndrome (AGS) [1, 4, 5, 8] (Fig. 5A). Genes with disease-causing mutations in AGS include the repair exonuclease TREX1 (3’ repair exonuclease 1); RNASEH2 (ribonuclease H2) subunits A, B and C; the Sterile Alpha Motif (SAM) domain and Histidine-Aspartate (HD) domain-containing protein 1 (SAMHD1); the double-stranded RNA-specific adenosine deaminase enzyme ADAR1; and the double-stranded RNA sensor Melanoma Differentiation-Associated Protein 5 (MDA5), encoded by Interferon Induced with Helicase C Domain 1 (*IFIH1*) [94, 95]. Patients with AGS share some clinical features with jSLE (including malar rash, chilblains, etc.) [96–98], but additionally exhibit severe neuropathology, cerebral calcifications (resembling the picture of congenital viral disease) and neurodegeneration [99].

While JAK inhibition controls systemic inflammatory features, they may be less efficacious in type 1 interferonopathies and patients with neuropsychiatric jSLE that is primarily driven by increased type 1 IFN expression [100]. Indeed, individual AGS patients on this treatment still developed neurological symptoms and/or neurological deterioration, and ataxia remained in patients with complement deficiency and an SLE phenotype after treatment with the JAK inhibitor baricitinib [94]. The reasons for this remain currently unknown, but low CSF titres of JAK inhibitors when compared to serum levels may play a role. Alternative treatments may include inactivation using IFN antibodies (sifalimumab, rontalizumab), type 1 IFN receptor blocking antibodies (anifrolumab) [101] or strategies to prevent the accumulation of cytoplasmic nucleic acids [102]. In AGS and autoimmune diseases, including SLE, activation of usually silenced endogenous retroviral elements has been proposed. Reverse transcription inhibition with nucleoside analogue reverse-transcriptase inhibitors has therefore been proposed and used in individual AGS patients (abacavir, lamivudine, zidovudine) [102] (Fig. 5C).

Considering observations from several studies reporting individual patients with “genetic” JSLE or lupus-like disease as well as recent findings from the UK jSLE Cohort Study [1, 4, 5, 8, 94–99, 101, 102], among patients with NP-jSLE, especially those with CNS involvement, we propose two main subgroups: (i) a chronic progressive, predominantly type 1 interferon-driven form that poorly responds to currently used treatments, and (ii) an acutely aggressive form that usually presents early during the disease that may be primarily mediated by auto-reactive effector lymphocytes. While this hypothesis requires to be tested in large collaborative international cohort studies, it may offer a basis for future patient stratification and individualised care.

## Conclusions

The lack of reliable clinical data, diagnostic strategies and evidence-based treatment options for NP-jSLE is striking, even for a rare complex disease such as jSLE. Because of the rarity of NP-jSLE and its variable presentation (19 suggested items) and disease course (acute, chronically progressing), national and international collaborations are necessary to define disease subgroups, and test phenotype-appropriate therapeutic interventions. Separation of acute, likely adaptive immunity-driven manifestations from chronic and likely type 1 interferon-mediated NP symptoms, may be a strategy towards individualised and target-directed care.

## Data Availability

Not applicable.
